# Transcriptome Analysis of Canine Cutaneous Melanoma and Melanocytoma Reveals a Modulation of Genes Regulating Extracellular Matrix Metabolism and Cell Cycle

**DOI:** 10.1038/s41598-017-06281-1

**Published:** 2017-07-25

**Authors:** Chiara Brachelente, Katia Cappelli, Stefano Capomaccio, Ilaria Porcellato, Serenella Silvestri, Laura Bongiovanni, Raffaella De Maria, Andrea Verini Supplizi, Luca Mechelli, Monica Sforna

**Affiliations:** 1 Department of Veterinary Medicine, 06126 Perugia, Italy; 2 Faculty of Veterinary Medicine, 64100 Teramo, Italy; 3 Department of Veterinary Sciences, 10095 Grugliasco (TO), Italy; 4 Present Address: Department of Pathobiology, Faculty of Veterinary Medicine, Utrecht University, Utrecht, The Netherlands

## Abstract

Interactions between tumor cells and tumor microenvironment are considered critical in carcinogenesis, tumor invasion and metastasis. To examine transcriptome changes and to explore the relationship with tumor microenvironment in canine cutaneous melanocytoma and melanoma, we extracted RNA from formalin-fixed, paraffin-embedded (FFPE) specimens and analyzed them by means of RNA-seq for transcriptional analysis. Melanocytoma and melanoma samples were compared to detect differential gene expressions and significant enriched pathways were explored to reveal functional relations between differentially expressed genes. The study demonstrated a differential expression of 60 genes in melanomas compared to melanocytomas. The differentially expressed genes cluster in the extracellular matrix-receptor interaction, protein digestion and absorption, focal adhesion and PI3K-Akt (phosphoinositide 3-kinase/protein kinase B) signaling pathways. Genes encoding for several collagen proteins were more commonly differentially expressed. Results of the RNA-seq were validated by qRT-PCR and protein expression of some target molecules was investigated by means of immunohistochemistry. We hypothesize that the developing melanoma actively promotes collagen metabolism and extracellular matrix remodeling as well as enhancing cell proliferation and survival contributing to disease progression and metastasis. In this study, we also detected unidentified genes in human melanoma expression studies and uncover new candidate drug targets for further testing in canine melanoma.

## Introduction

Despite recent advances in the understanding of its pathogenesis, melanoma remains the deadliest form of malignant skin tumor in human beings and its annual incidence has increased by more than 60 percent from 1991 to 2011 (http://www.cancer.gov/research/progress/snapshots/melanoma). No effective therapy is currently available and tumor-associated mortality is due to metastatic spread to distant sites^1^. In human medicine, several studies have identified different patterns of gene expression in melanoma cells compared to normal melanocytes and between different stages of melanoma progression^2, 3^. However, in recent years, numerous studies have demonstrated that not only genetic and epigenetic changes of neoplastic cells are important to contribute to the malignant potential of a tumor but tumor microenvironment plays an important role in determining the potential for tumor invasion and metastasis. Cancer associated fibroblasts (CAFs) are one of the principal cell types of the stromal compartment and play a role in tumor cell migration through the stroma^4, 5^. Tumor cells activate CAFs by secreting various cytokines and by undergoing epithelial-mesenchymal transition; CAFs, on their side, exert a tumor-promoting function through direct (paracrine soluble factors and cell-to-cell contact) and indirect (modulation of other stromal components) approaches^6, 7^. In epithelial tumors, CAFs help neoplastic cells to undergo the epithelial-mesenchymal transition and acquire a phenotype that is optimal for migration and invasion at distant sites^6, 8, 9^ and possibly confers chemoresistance^10^. Less is known about the function of fibroblasts in non-epithelial tumors such as melanoma, but evidence suggests similar roles in tumor development and progression. CAFs induced by melanoma cells produce proinflammatory mediators and proteases, favor resistance to apoptosis and regulate the anti-tumor response, creating a microenvironment that facilitates the proliferation, survival, invasion and metastasis of the tumor^11–15^. Therefore a bidirectional interdependency seems to be fundamental in promoting tumor invasiveness and aggressiveness^16^ and a multidirectional approach is essential when new anti-cancer strategies are planned and developed. The use of RNA sequencing (RNA-seq) technology has expanded the diagnostic, prognostic and therapeutic opportunities in cancer research, allowing the recognition of gene fusions and differential expression of disease-associated transcripts^17, 18^. In the melanoma field, many studies have focused on RNA-seq of melanocytes^19^ and melanoma cell lines^20–22^ and only very recently few of them on whole tumor samples or single malignant, immune, stromal and endothelial cells^18, 23–26^. In veterinary medicine, this technique has been used in recent years to study degenerative diseases and stress-related conditions and only rarely has it applied to tumor analysis in canine head and neck carcinoma, mammary carcinoma and lymphoma^27–30^. To the author’s knowledge, no RNA-seq studies have been conducted in veterinary medicine to investigate the differentially expressed genes and to elucidate the interactions between neoplastic cells and stroma in canine cutaneous melanoma and melanocytoma. The dog is considered a valid model for several human cancers^31, 32^ and for human melanoma because these tumors in dogs share the same locations as in humans (skin, mucosal sites, nail matrix and eyes)^33, 34^. However, despite some similarities, the pathogenesis of canine melanoma has not been fully elucidated and the validity of the canine model to study human melanoma is still under debate. Regarding the cutaneous tumors, dogs are likely to have different risk factors compared to humans, where cutaneous melanomas are mostly UV-induced^34^. Therefore, the aim of our study was to analyze, through RNA-seq analysis, the differential gene expression of canine cutaneous melanocytomas and melanomas. Results were validated through qRT-PCR and immunohistochemistry. Due to the importance of the tumoral microenvironment and the reciprocal influence between neoplastic and stromal cells, we used RNA extracted from formalin-fixed, paraffin-embedded whole primary tumor samples, avoiding the use of cell lines. A total of 8 cases (4 melanocytomas and 4 melanomas) were retrospectively selected for the purpose of this study. As there are no universally accepted criteria to prognosticate canine cutaneous melanocytic neoplasms, for the RNA-seq analysis we selected melanomas that had proof of their malignant behavior through the metastatic involvement of regional lymph nodes or distant organs. To confirm the results of the RNA-seq, we also investigated the mRNA expression of some target genes on a larger series of samples through qRT-PCR. Immunohistochemical analysis was used to study the protein expression of two differentially expressed genes. We hypothesized that benign melanocytic tumors and malignant tumors and tumors with proven metastatic abilities would show a differential gene and protein expression pattern and that the genes/proteins involved in the metastatic signature would belong to pathways revealing a relationship between neoplastic cells and tumor microenvironment.

## Results

### Clinical data and histological examination

Data regarding the signalment, location and diagnosis of the tumor are summarized in Table 1. The age of affected dogs ranged from 3 to 14 years with an average of 9 years. Eight cases were females, fifteen were males and for two animals the gender was unknown. Benign tumors were mostly located in the eyelid whereas malignant tumors where more commonly located on the distal extremities and digits. Histological re-examination confirmed the original diagnosis of melanocytomas and melanomas in all cases. The amount of stroma in malignant tumors was slightly higher than in benign tumors (mean percentage of tumor stroma in melanomas = 26%; mean percentage of tumor stroma in melanocytomas = 20%).

**Table 1:** Tumor location, signalment, percentage of tumor stroma and type of analysis performed in affected dogs.

Case	Diagnosis	Breed	Age (Y)	Gender	Location	Metastasis	% stroma (% tumor area)	Examined by
1	Melanoma	Mixed	10	M	—	Regional lymph node	15	RNA-seq, qRT-PCR, IHC
2	Melanoma	Giant Schnauzer	11	M	Digit	Popliteal lymph node	45	qRT-PCR, IHC
3	Melanoma	Labrador	8	M	Digit	Popliteal lymph node	30	qRT-PCR, IHC
4	Melanoma	Mixed	11	F	Perianal	Lung	20	RNA-seq, qRT-PCR, IHC
5	Melanoma	Mixed	5	M	Maxilla	Lung, brain	40	RNA-seq, qRT-PCR, IHC
6	Melanoma	Dachshund	8	F	Abdomen	Inguinal lymph node	20	RNA-seq, qRT-PCR, IHC
7	Melanoma	German Shepherd	8	—	Lateral thorax	None suspected	15	qRT-PCR, IHC
8	Melanoma	Labrador retriever	14	M	Digit	None suspected	30	IHC
9	Melanoma	Rottweiler	11	M	Digit	None suspected	25	qRT-PCR, IHC
10	Melanoma	Miniature pinscher	6	M	Limb	None suspected	40	qRT-PCR, IHC
11	Melanoma	Miniature pinscher	11	F	Digit	None suspected	25	qRT-PCR, IHC
12	Melanoma	Mixed	9	FS	Ear	None suspected	15	qRT-PCR, IHC
13	Melanocytoma	Alaskan husky	13	MC	Eyelid	None suspected	5	RNA-seq, IHC
14	Melanocytoma	Boxer	10	M	Eyelid	None suspected	15	qRT-PCR
15	Melanocytoma	Pinscher	9	FS	Muzzle	None suspected	40	qRT-PCR, IHC
16	Melanocytoma	Pointer	—	M	Eyelid	None suspected	10	RNA-seq, qRT-PCR, IHC
17	Melanocytoma	Mixed	11	FS	Lateral thorax	None suspected	5	RNA-seq, qRT-PCR
18	Melanocytoma	German Shepherd	9	M	Lateral thorax	None suspected	20	RNA-seq, qRT-PCR, IHC
19	Melanocytoma	Mixed	4	M	Chin	None suspected	10	qRT-PCR, IHC
20	Melanocytoma	Irish setter	—	—	Chest	None suspected	20	IHC
21	Melanocytoma	Mixed	9	FS	Muzzle	None suspected	10	qRT-PCR, IHC
22	Melanocytoma	Mixed	10	M	Eyelid	None suspected	20	qRT-PCR, IHC
23	Melanocytoma	Giant Schnauzer	10	F	Limb	None suspected	30	IHC
24	Melanocytoma	Golden Retriever	3	M	Lip	None suspected	35	IHC
25	Melanocytoma	German Shepherd	7	M	Lip	None suspected	40	IHC

### RNA Sequencing and bioinformatic analysis

**Figure 1:**
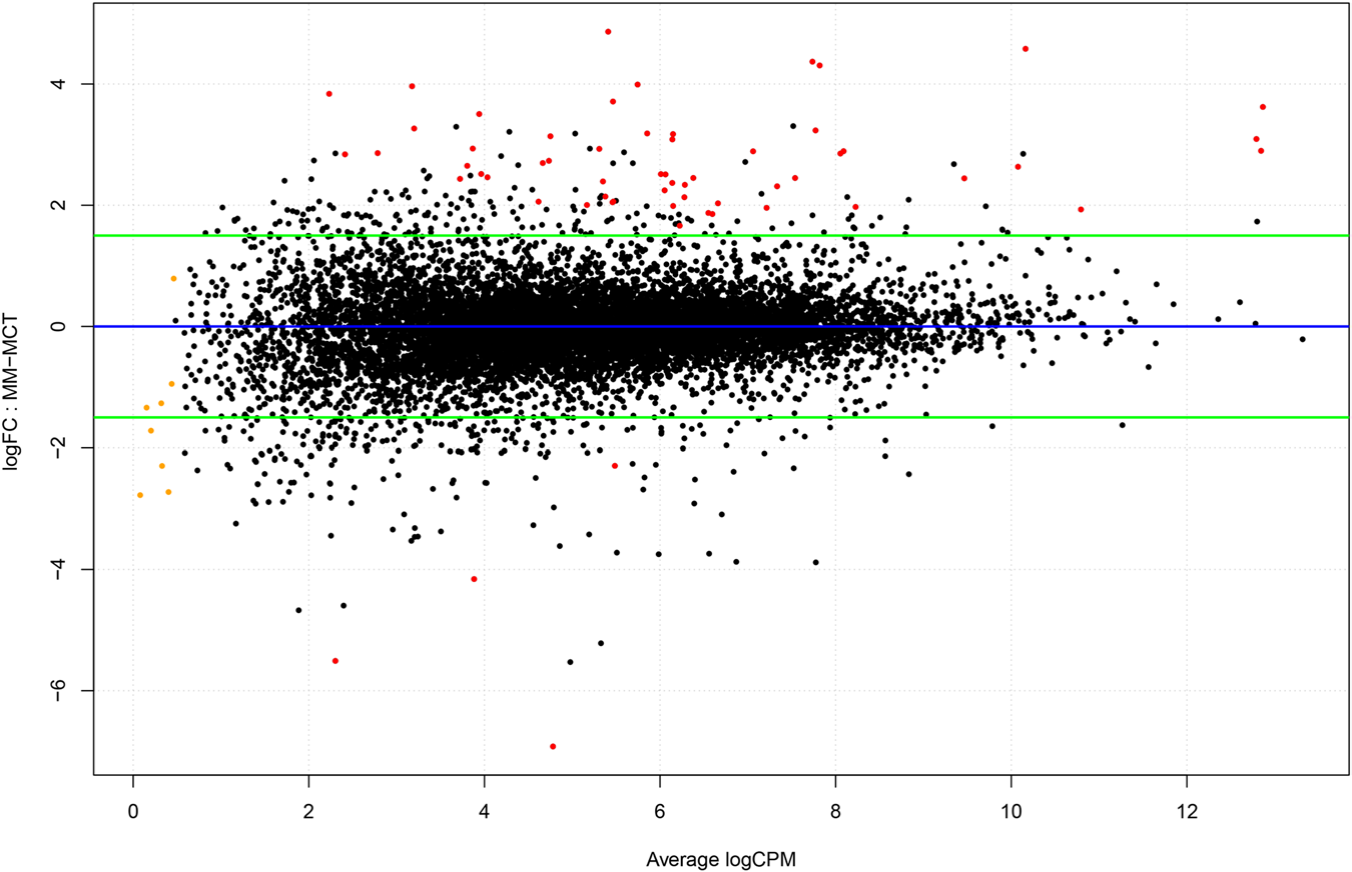
MA plot showing the relationship between average concentration (logCPM) and fold-change (logFC) across the genes. Each gene is represented by a black dot. Significant differentially expressed genes are colored in red. The orange dots represent genes in which the counts were zero in all samples of one of the groups. The green lines represent logFC +/− 1.5 threshold.

**Figure 2:**
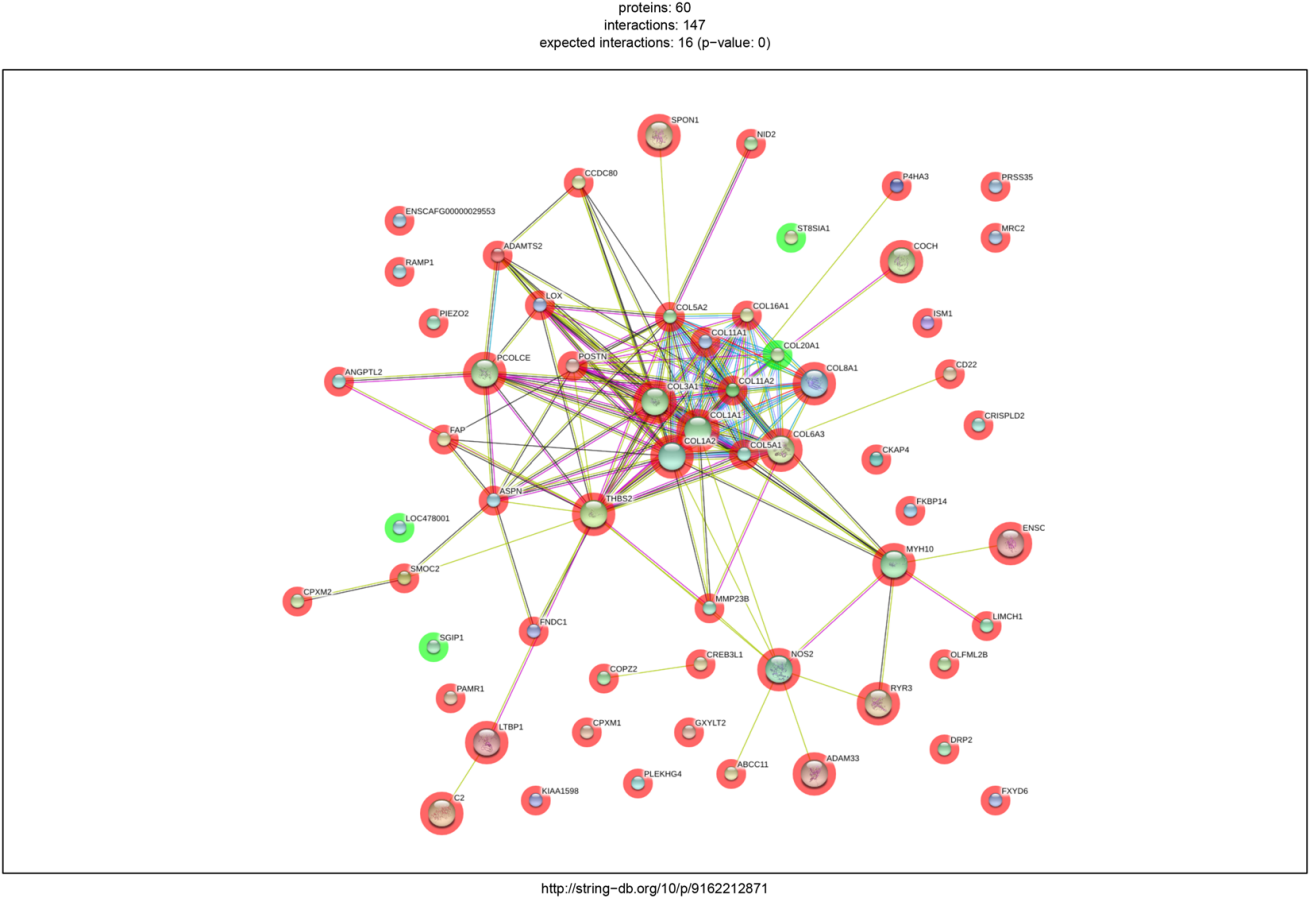
Network of the differentially expressed genes produced by String. Circles are proteins while lines are interactions. Known interactions are represented in purple (experimentally determined) and azure (from curated databases). Red, green and blue lines pertain predicted interactions (respectively gene neighborhood, fusions and co-occurrence) while yellow, indigo and black are interactions found from other sources (text mining, co-expression and protein homology). The red halo around gene represents up-regulation while the green one down-regulation.

Sequencing on Illumina platform produced 288 million read pairs, distributed as detailed in Supplementary Table S1. The mean number of reads per sample was over 36 millions. Quality control and trimming procedures retained over the 99% of the produced sequences. Percentage of uniquely mapped reads on the reference genome was variable throughout the samples ranging from unacceptable quotas around 15% up to 93% of the cleaned sequences. In this step we excluded the two samples that had too low mapping statistics (sample n. 4 and n. 5). The low percentage of uniquely mapped reads to the reference genome strongly suggested that library preparation was compromised. Indeed, exploratory analysis revealed that these 2 samples had to be excluded due to bad clustering and overdispersion of sequence data: the final dataset consisted of six libraries (4 melanocytomas – n. 13, 16, 17 and 18 – and 2 melanomas – n. 1 and 6). Uniquely mapped reads only (sequences matching only one position throughout the entire genome) were used for the differential gene expression assessment to avoid introducing bias through multi-mapper assignment uncertainty. After statistical analysis with edgeR, where 11,783 genes were “expressed” (Count per Million >1 in at least half the samples), we found 60 differentially expressed genes in melanoma with respect to melanocytoma at a significance q < 0.05 and absolute fold change (logFC) equal to 1.5. With these filters, 56 genes resulted up-regulated (logFC > 1.5) while 4 genes were down-regulated (logFC > −1.5) (Fig. 1 and Supplementary Table S2). After annotation of the modulated transcript list through BioMart, the gene names retrieved were used to perform enrichment analysis. The annotated list from the differential gene expression analysis was used as input for the pathway analysis. The enrichment according to the three biological vocabularies of “Gene Ontology” (Cellular Component, Biological Process and Molecular function) was assessed using the tools available on String-DB (Search Tool for the Retrieval of Interacting Genes/Proteins). The analysis carried out on the DGE has produced a network with large number of highly interconnected genes particularly with experimentally determined interactions (Fig. 2). All the 60 differentially expressed genes had the corresponding protein match (node) leading to 146 interactions between nodes (edges). The expected value for this subset was 16, way lower than the observed, and p-value for these enrichment considering the genomic background resulted to be 0 (Supplementary Table S3, Sheet 1). After highlighting the highly interconnected network, we focused on pathways and biological processes enriched (FDR < 0.05) with molecules composing the network itself. The significant pathways identified in KEGG (Kyoto Encyclopedia of Genes and Genomes: collection of databases that is genomic data, biological pathways, diseases, drugs and chemicals questioned by String-DB) involve extracellular-matrix (ECM) interaction, focal adhesion, PI3K-Akt signaling pathway, and platelet activation, as detailed in Table 2. The enrichment is even more compelling in the three GO vocabularies, using the same set of genes against the annotated human genome (Supplementary Table S3, Sheet 4 to 6). For example in the “biological process” section: extracellular matrix organization, collagen catabolic process, extracellular matrix disassembly and in the “molecular function” section: extracellular matrix structural constituent, metallopeptidase activity and cell adhesion molecule binding.

**Table 2:** Significantly enriched KEGG pathways according to StringDB.

Pathway ID	Pathway description	Observed gene count	FDR
4512	ECM-receptor interaction	9	1.22e-09
4974	Protein digestion and absorption	8	1.82e-08
5146	Amoebiasis	8	8.33e-08
4510	Focal adhesion	9	6.27e-07
4151	PI3K-Akt signaling pathway	10	2.22e-06
4611	Platelet activation	7	4.1e-06

### qRT-PCR

**Figure 3:**
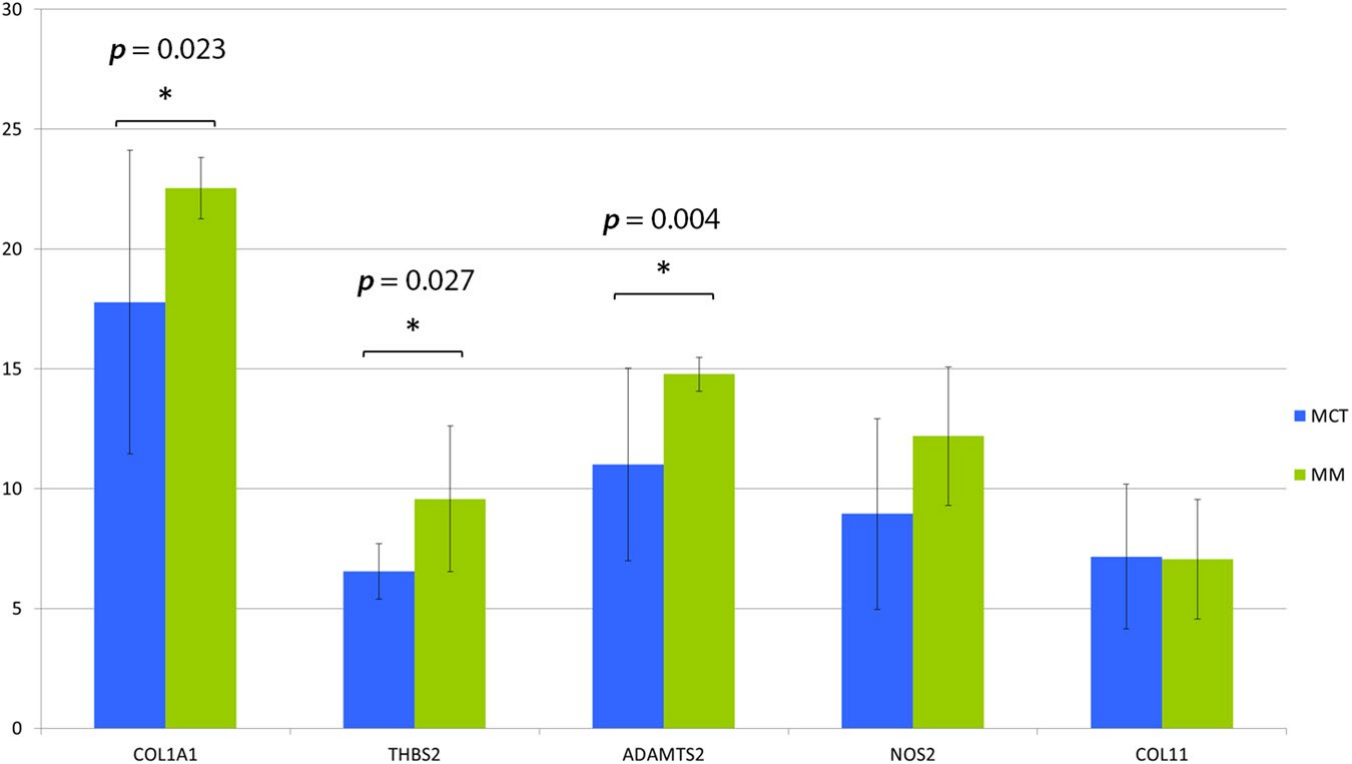
Bar charts representing relative expression values of *COL1A1*, *THBS2*, *ADAMTS2*, *NOS2* and *COL11* genes in melanocytomas (blue) and melanoma (green) on a base 2 logarithmic scale. Whiskers define a 95% CI (confidence interval). Statistical significance is evidenced with asterisks and relative *p*-value.

geNorm provides a ranking of the tested genes, considering their expression stability, selecting reference genes according to the stability measure M (average pairwise variation of each gene against all others). Both *HBS2* and *GUSβ* genes displayed a relatively high stability with M values of 0.4, far below the accepted limit of 1.5^35^. *THBS2*, *COL1A1*, *ADAMTS2* resulted significantly up-regulated in the group of melanomas compared to melanocytomas (P < 0.05, Mann–Whitney). *COL11A1* and *NOS2*, although slightly unregulated, did not show a statistically significant difference between the two groups of tumors (Fig. 3).

### Immunohistochemical examination

**Figure 4:**
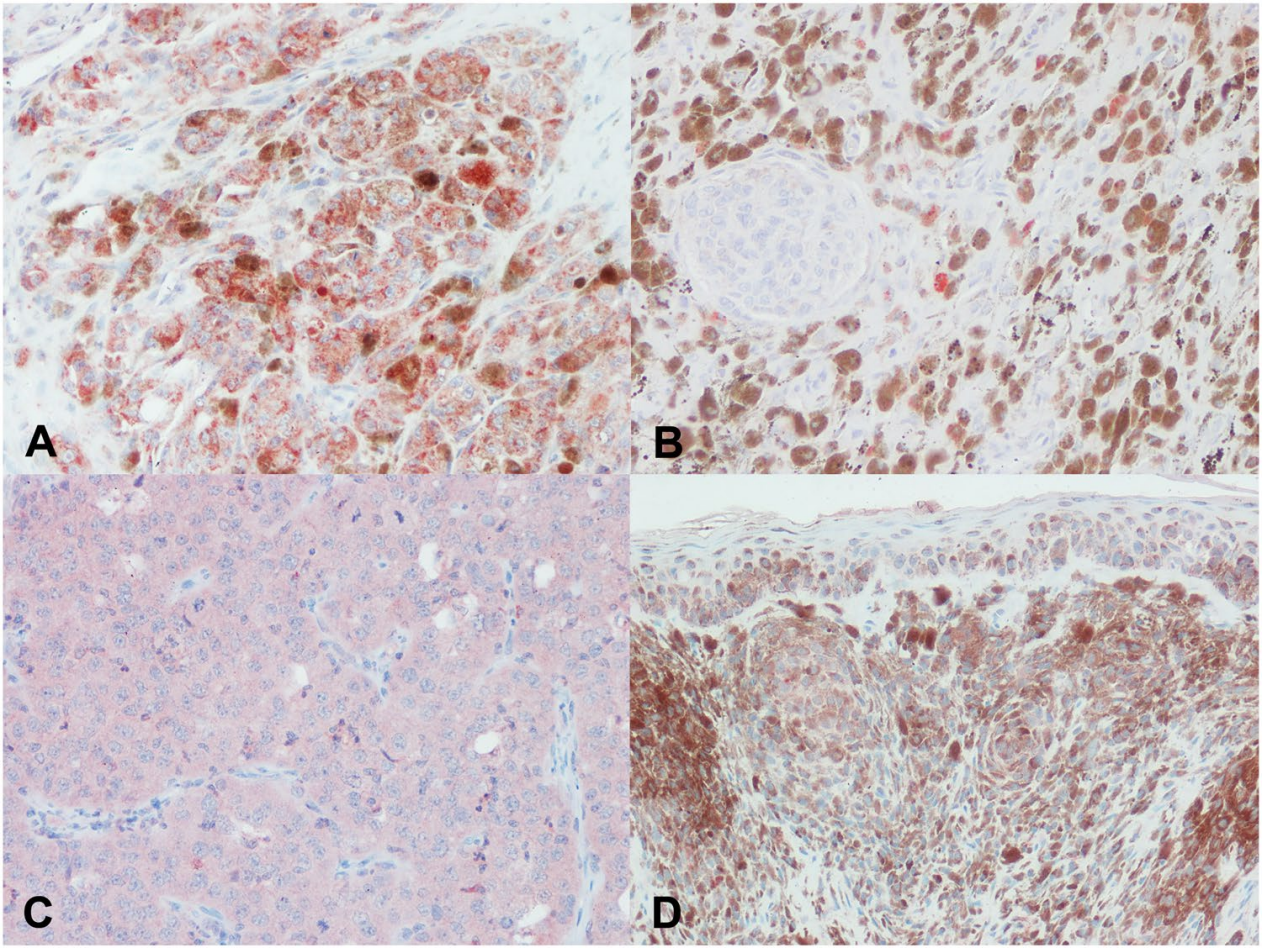
Immunohistochemical expression of COL1 and THBS2 in melanocytic tumors. (**A**) Cytoplasmic positivity for COL1 in numerous neoplastic cells and fewer stromal cells in a melanoma; (**B**) occasional COL1 positive cells in melanocytoma; (**C**) diffuse cytoplasmic stain for THBS2 in an amelanotic melanoma; (**D**) scattered and mild THBS2 positivity in melanocytoma cells.

Cytoplasmic reactivity for COL1 was observed both in neoplastic and stromal cells in melanomas and melanocytomas, whereas THBS2 was expressed only by neoplastic cells (Fig. 4). The average percentage of positive cells for COL1 was 86.67% in melanomas and 72.73% in melanocytomas (P = 0.08), whereas for THBS2 was 64.17% in melanomas and 54.27% in melanocytomas (P = 0.44). No statistically significant differences were noted between the two groups in terms of percentage of positive cells, intensity of immunoreactivity, pattern of reaction and distribution of reactivity.

## Discussion

**Figure 5:**
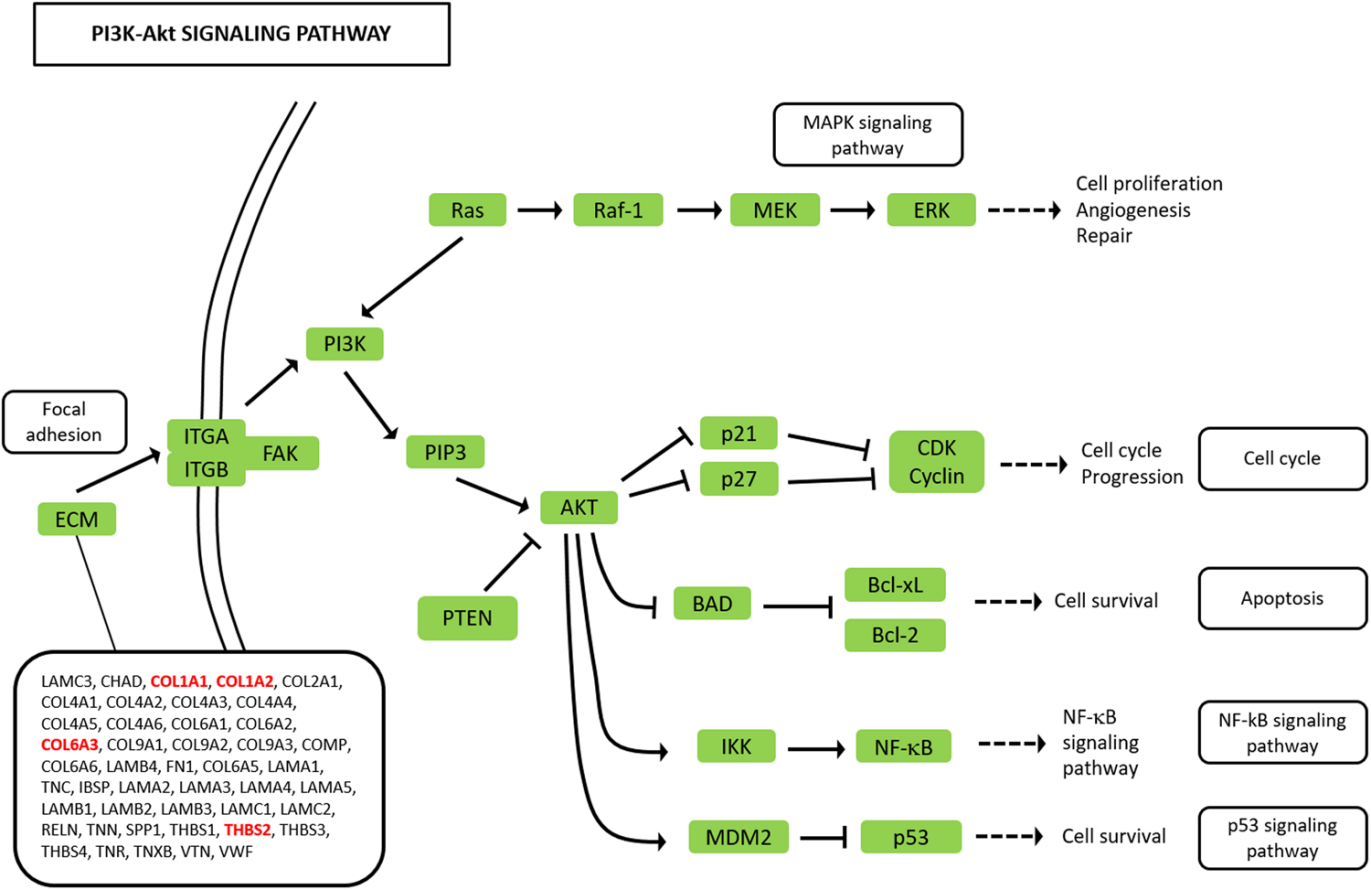
The extended Kegg Pathway for PI3K-Akt signaling. Genes activating the ECM-receptor interaction pathway and upstream of the PI3K-Akt molecule are depicted in the rectangle. The genes that were differentially expressed in this study are bold face and red colored.

Our RNA-seq analysis study confirmed the presence of differentially expressed genes in canine cutaneous melanocytomas and melanomas. The differentially expressed genes belong to the PI3K-Akt activation pathway that has already been demonstrated to be activated in human and canine melanomas^36–38^. The KEEG pathway analysis revealed a link between the PI3K-Akt pathways and the extracellular environment were the focal adhesion and the ECM pathways are upstream regulators of the PI3K-Akt (Fig. 5). Our results also suggest the activation of newly detected genes involved in the ECM-cell interaction pathway that have not yet identified in human melanoma expression studies. The RNA-seq approach has been uncommonly used to study human melanoma cell lines^18, 21, 39^ and only single studies have examined human primary and metastatic melanoma tumor samples^23^. Numerous authors have demonstrated a reciprocal effect of host-tumor interactions on neoplastic and stromal cells and a synergistic effect of co-cultivation of human melanoma and stromal fibroblasts in invasive and metastatic abilities^11, 16, 40–42^. These results have substantiated the observation that data obtained from studies based on melanoma cell lines and melanocyte cultures may not always be applicable to melanocytic tumors and their *in vivo* biology. Therefore, our goal was to investigate the whole neoplastic tissue, avoiding the use of cell cultures, in order to explore the interactions between cancer cells and stromal components in canine melanocytic tumors. To our knowledge, no similar approach has been used on canine tissues. In a previous experiment, we attempted at extracting RNA from lentigo (melanocytic hyperplasia) lesions from 4 dogs that would have served as normal melanocyte control. However, the yield of RNA was insufficient for further analysis and therefore our study focused on the differential expressed genes in benign (melanocytomas) and malignant (melanomas) tumors. The inclusion criteria for the latter in the RNA-seq was that the melanomas must have had proof of metastatic behavior through a histologically confirmed lymph node metastasis or systemic dissemination, since the currently used histologic criteria are partially insufficient to predict the prognosis of canine cutaneous melanomas^43^. The comparison of gene expression profiling between metastatic melanoma and melanocytoma cases identified 60 genes with a differential expression: 56 genes were up-regulated and 4 were down-regulated. The difference in gene expression was almost always more than two logFC compared to the melanocytoma group. This strong difference suggests an activation of differential pathways associated with cancer progression in melanomas. The enriched pathway analysis revealed that the differentially expressed genes involve genes regulating cell survival and proliferation and in particular the PI3K-Akt pathway. Our results are similar to studies conducted on canine melanoma tumors and melanoma cells lines which have demonstrated that the PI3K-Akt pathway is dysregulated in the dog, with expression patterns similar to human melanomas^44, 45^. On the contrary, *BRAF* and *NRAS* mutations appear to be uncommon in canine melanomas^34, 44, 46–48^ and our results, although based on gene expression profiling, let us speculate that mutations activating these genes are indeed rare in canine cutaneous melanoma. In addition to the PI3K-Akt pathway, the gene expression analysis of our case series demonstrated a strong upregulation of ECM-receptor interaction pathway in melanomas compared to melanocytomas. Many of the genes involved are indeed upstream regulators of the PI3K-Akt pathway, through the link of focal adhesion family proteins, such as integrins. The upregulation of this pathway is also supported by the differentially expressed *NOS2* (nitric oxide synthase 2, inducible NOS – iNOS). *NOS2* has been demonstrated to favor the metastatic potential of melanoma, either directly modulating tumor cell metabolism^49^ and driving survival and proliferation of human melanoma cells^50^ or indirectly through the activation of pro-tumorigenic γδ T cells^51^. Our results are further supported by a very recent study which demonstrates that human melanoma cells co-cultured in the presence of fibroblasts undergo a phenotype switch toward mesenchymal-like cells, activate the PI3K-Akt/mTOR signaling pathway and become resistant to the BRAF inhibitor vemurafenib^52^. Our study also reveals an overexpression of fibronectin type III domain, a mechanically sensitive protein forming interacting fibrils in the extracellular matrix. This finding is in accordance with a study by Cho and colleagues that elegantly demonstrates that fibronectin type III domain is able to induce resistance to apoptosis in human lung cancer cells, through the activation of the PI3K-Akt/αvβ5 signaling axis^53^. *ADAMTS2* (a disintegrin and metalloproteinase with thrombospondin type 1 motif 2) was overexpressed in melanomas, compared to melanocytomas. Giricz *et al*. demonstrated an overexpression of *ADAMTS2* in melanoma tissues of affected individuals compared to normal tissue of the same patients^54^. In the same study, the expression of this gene was also investigated in metastatic melanoma cell lines compared to cultured fibroblasts and was found to be higher in fibroblasts compared to metastastic melanoma cells. This would be consistent with its role in pro-collagen processing. Therefore, we suggest that the ECM-receptor interaction pathway could represent an alternative pathway contributing to the downstream activation of PI3K-Akt in canine cutaneous melanomas. To further confirm our results, a qRT-PCR analysis was performed on a larger series of samples of melanomas and melanocytomas. A statistically significant difference between melanomas and melanocytomas was observed for *COL1A1*, *THBS2* and *ADAMTS2*. Although slightly up-regulated in melanomas, *COL11A1* and *NOS2* did not show a statistically significant difference. This could be due to the low number of samples or to the high individual variability. The immunohistochemical protein expression analysis could be performed on COL1 and THBS2 only due to the unavailability of specific anti-canine antibodies for FFPE tissues. The results of this analysis revealed a higher expression of both markers in melanomas compared to melanocytomas, yet the difference was not statistically significant. As immunohistochemistry is not a quantitative technique and is less sensitive than PCR, the low number of samples could have impacted on the significance of the results.

No ECM/collagen pathway activation has ever been demonstrated before in melanomas, despite numerous ECM-related genes have been associated with poor clinical outcomes in several types of cancer^55–59^. In primary human breast carcinomas, different sets of ECM-related genes have been confirmed to have an impact on the clinical outcome of the tumor^60^. However, no differences in the expression of their corresponding products were noted in the same study and no correlation was found between the differentially expressed EMC genes and different features of the stroma. This can be due to post-translational modifications of ECM components^61^ or to the need for specific spatial distribution of mutually interacting compounds that is necessary for the activation of EMC related enzymes^62^. In this view, in recent years a new concept has emerged regarding the “matrisome”, defined as the ensemble of genes encoding ECM and ECM-associated proteins, as a tool to better characterize the ECM composition, metabolism and biology in order to gain new insight on prognostic and diagnostic markers and therapeutic opportunities^63^. Among the extracellular matrix-receptor interaction pathway, several collagen genes were upregulated in malignant melanomas compared to melanocytomas; one of the most upregulated was *COL11A*. A collagen-remodeling gene signature has been found in human ovarian cancers, where levels of COL11A1 continuously increase during disease progression, with the highest expression in recurrent metastases^59^. In the same study, other genes such as *COL5A1*, periostin (*POSTN*), thrombospodin 2 (*THBS2*) and *LOX* were upregulated and associated with poor survival after adjuvant chemotherapy; these genes were also upregulated in our melanoma case series. Another study found that the expression of *COL11A1* and *COL5A2* genes in colorectal cancers is correlated with carcinoma aggressiveness and progression, as well as lymph node metastasis, and that these genes are not expressed in normal colon samples^64^. *COL11A1* has also been found to be expressed at the front of invasive carcinomas by presumably carcinoma cells, putatively undergoing epithelial-to-mesenchymal (EMT) transition; also, carcinoma-derived cells with highly metastatic capabilities can express *COL11A1* ^65^. To the author’s knowledge, there are no studies investigating or demonstrating the role of *COL11A1* in melanomas, neither in human nor veterinary literature; however, as molecular equivalents of EMT have been demonstrated in human melanomas as well^66^, it is reasonable to think that a similar process involving *COL11A1* is taking place in melanoma. In deeply invasive human cutaneous melanomas, *COL1* mRNA was found to be strongly expressed in stromal cells close to and encircling melanoma nests in the papillary dermis, whereas this expression was not noted in the deep fibroblasts in the reticular dermis, demonstrating the presence of two different microenvironments in cutaneous melanomas. Inhibition of *COL1* expression before tumor development in an experimental porcine model decreased the invasivity of the tumors by decreasing the number of tumor-associated blood vessels, without affecting melanoma cell proliferation^67, 68^. Therefore, *COL1* expression by tumor associated fibroblasts can have a proangiogenic effect, where *COL1* can be important for supporting and guiding the developing endothelial cells. In our study, we did not investigate the tumor vasculature. However, *COL1A1* and *COL1A2* were both strongly overexpressed in the melanomas compared to melanocytomas, in agreement with the study from Van Kempen. Furthermore, collagen type I and type III have been demonstrated to be able to down-regulate the expression of E-cadherin in pancreatic cancer cell lines^69^ and it is known that the down-regulation of E cadherin is an important step in melanoma progression^66^. In our immunohistochemical analysis, COL1 expression was found not only in fibroblasts of the stroma of the tumor but also in the neoplastic cells. Therefore, collagen production by neoplastic cells could favor E cadherin down-regulation and the epithelial-to-mesenchymal transition. Our study also showed an overexpression of thrombospondin-2 in melanomas compared to melanocytomas. Thrombospondin-2 is considered a potent endogenous inhibitor of tumor growth and angiogenesis^70^. This molecule has also been proven to suppress hematogenous metastasis in human malignant melanoma cell lines through microenvironment-modifications such as increase of the PA (plasminogen activator) inhibitors and decrease of the vascularity of metastatic lesions^71^. However, other studies demonstrated a differential expression of this molecule between melanoma metastases and primary tumors, suggesting that the modulation of cell-matrix interactions is pivotal in the pathogenesis of malignant melanoma metastasis^72^. Differences in these results could be explained by mechanisms causing a rapid degradation of mRNA or blocking its translation into a protein or by the instability of the protein, which appears to be very unstable once it is secreted, as is the case for the other members of the thrombospondin family^73^. Worthy of note is also the upregulation of *LOX* in our malignant melanoma samples. The LOX family proteins have been long known to modulate extracellular matrix molecules, promoting a migratory, highly invasive and metastatic phenotype in human breast cancer cell lines as well as in rat prostatic cell lines and human cutaneous melanoma cell lines^74^. In the same study, endogenous *LOX* mRNA expression could be induced in poorly invasive breast cancer cell lines by cultivating cells in the presence of fibroblasts-conditioned medium or matrix, suggesting a role for stromal fibroblast in LOX regulation. Furthermore, LOX proteins have also been shown to have intracellular functions such as direct or indirect Snail activation, the latter mediated by hypoxia-inducible factor 1alpha (HIF-1alpha) recruitment^74, 75^. By comparing the morphological features (hematoxylin and eosin, H&E) of our samples, the melanomas were characterized by a slightly more prominent stroma than the melanocytomas. Therefore, we cannot exclude that the differential expression of collagen and ECM genes found in our study is due to a different tumor-to-stroma ratio and to a difference in the amount of collagen tissue between the two groups. However, the difference between the two groups was not marked and, due to the same tissue origin (skin) for both sample groups, the more abundant connective tissue in melanomas is considered as a melanoma-related effect. The stroma, in response to signals most likely originating from transformed cells, can be therefore indirectly responsible for the generation of a microenvironment that is promoting cell-matrix interactions and connective tissue remodeling, thereby facilitating a prometastatic environment. The identification of collagen-remodeling genes as a metastatic and poor prognosis gene signature suggests that collagen remodeling might be a common biologic process that contributes to poor overall survival (OS) among dogs with cutaneous melanomas. Our RNA-seq experiment was done on a small case series, due to the rarity of canine cutaneous melanomas with proven malignant behavior. A larger scale study with a higher number of metastatic tumors would be worthy. Finally, in our study we used FFPE samples: this treatment is known to produce bias, as we directly observe in some of our libraries and probably affecting low expressed genes due to high rate of RNA degradation. However, studies have been conducted^76^ proving that results on FFPE samples correlated with the one obtained with fresh tissue with some caveats.

In conclusion, in the present study, we characterized the transcriptome of melanoma vs melanocytoma samples, with cutting edge techniques, in the canine model species where no such data are available. The RNA-seq data were confirmed with qRT-PCR and immunohistochemistry. Novel is also the choice of whole paraffin sections instead of tumor cell primary cultures, which is considered more representative of the tumor biology *in vivo* and of the interactions between tumor cells and stroma. This study shows that gene expression levels in melanomas were different from melanocytomas, indicating an association with malignancy. The analysis of disease process categories showed that canine melanoma shares activation of similar molecular pathways as human melanoma, such as PI3K-Akt. However, most likely this pathway is activated by different upstream mechanisms, such as a strong upregulation of genes involved in collagen and extracellular matrix metabolism, organization and remodeling. We hypothesize that the developing melanoma actively promotes collagen metabolism, extracellular matrix remodeling, cell proliferation and survival mechanisms contributing to disease progression and metastasis. In order to define the biological and clinical significance of our findings, detailed molecular mechanistic studies are needed to determine the causal relationships of the differentially expressed genes and oncogenesis. These findings would support further investigations on the utility of therapeutic targeting of ECM-cell matrix interaction pathways in canine melanoma treatment. Finally, in this study, we also detect genes not yet identified in human melanoma expression studies. Comparative studies could then explore the translational potential of such approach for the treatment of the human disease.

## Methods

### Samples

Twelve formalin-fixed, paraffin-embedded (FFPE) samples (6 melanocytomas and 6 melanomas) were selected from the archives of our Departments. Samples had been trimmed and processed according to current recommended guidelines^77^. Melanoma samples that were submitted to RNA seq were chosen between the tumors that had proof of metastatic behavior (histologically confirmed lymph node metastasis/distant metastasis).

### Histological examination

Sections (4 μm thick) were cut from the paraffin blocks and stained with H&E. The tumors were evaluated blindly and independently by three experienced pathologists (CB, IP, MS). Tumors were reclassified using histologic parameters with the most statistically supported validity for prognostic use in canine melanocytic neoplasia, according to the recent classification^43, 78–80^. According to these criteria, all tumors that had concomitant lymph node/distant metastases had been correctly classified as malignant (“melanoma”). The three pathologists empirically calculated the ratio between stroma and tumor, considering the area represented by stromal tissue on the whole tumor mass and assigning it a percentage value.

### RNA extraction

RNA extraction was performed from four to six, 8 µm-thick paraffin sections with a commercial kit following the manufacturer’s instructions (RecoverAll Total Nucleic Acid Isolation Kit, AM 1975, Ambion, Austin, Texas). Residual genomic DNA was removed from the total RNA by DNAse treatment, which was included in the commercial kit. Normal tissue located at the tumor margins was carefully resected and discarded with the help of a scalpel blade or a sterile needle during the paraffin sectioning procedure. RNA quantity and quality were evaluated using NanoDrop 2000 spectrophotometer (Thermo Fisher Scientific, Waltham, MA, USA) and Qubit 2.0 Flurometer (Life Technologies, MA, USA), while RNA integrity was assessed by microfluidic electrophoresis on BioAnalyzer 2100 (Agilent Technologies).

### RNA sequencing

FFPE samples suitable for library preparation according to quality/quantity evaluation, were processed concurrently and following the manufacturer’s specifications with the Illumina TruSeq Stranded Total RNA (Ribozero) ver. 3 for a total of 8 samples, 4 derived from melanoma and 4 from melanocytoma cases. After rRNA depletion the samples were not fragmented because the RNA Integrity Number of starting material was very low. Massive parallel sequencing was carried out on an Illumina HiSeq 1500 machine generating 101 bases paired-end reads.

### Bioinformatic analysis

RAW sequences from the sequencer were checked for quality control and trimmed from adapters using FastQC (http://www.bioinformatics.babraham.ac.uk/projects/fastqc) and Trimmomatic v. 0.33^81^, respectively. After this step, the resulted paired reads were mapped using STAR v.2.4.0.1^82^ with default parameters, guided by the Ensembl v.84 transcript annotation downloaded from the UCSC Table browser, on the *Canis familiaris* reference genome (CanFam3.1). Common quality parameters in RNA-seq experiments (insert length, gene mapping bias, RNA junctions) were evaluated using RSeQC^83^. To account for differential expression between the groups, a count based approach using uniquely mapped reads only was used: aligned reads were classified as counts on genes with the software featureCounts using the Ensembl 84 annotation coordinates^84^ and gene expression changes identified using edgeR^85^. EdgeR package uses the negative binomial distribution as a model to analyse the differential gene expression from RNA-seq data between conditions, accepting as input a matrix with the raw counts number for each sample and each annotated feature. Briefly, a matrix were rows represented the Ensembl gene annotation and columns represented the sample was imported into R and differential gene expression was assessed following best practices for the edgeR package. A gene was considered differentially expressed if the False Discovery Rate (FDR) adjusted p-value (q-value) was lower than 0.05 and log Fold Change comprised between −1.5 and +1.5. Differentially expressed genes between two groups were annotated using BioMart (http://www.ensembl.org/biomart/martview/).

### Enrichment analysis

Different approaches were applied to search for enriched Gene Ontology (GO) terms and pathways. The differentially expressed gene list was used as input for STRING DB R Package^86^. STRING (http://www.string-db.org) is a database of known and predicted protein-protein interactions. The interactions include direct (physical) and indirect (functional) associations. The database collates information from different sources ranging from experimental evidences to library mining interactions providing confidence scores for each interaction.

### qRT-PCR assays

Total RNA (250 ng) of each sample was reverse transcribed in triplicate using the SuperScript® VILO™ Master Mix (Thermofisher), according to the manufacturer’s specifications and successively put together in a single aliquot. Successful reverse transcription was confirmed by PCR amplification of the Canis familiaris GUSβ gene (NM_001003191). Primers on reference genes (*GUS*β, *HMBS*) and on genes of interest (*THBS2*, *COL1A1*, *COL11A1*, *NOS2*, *ADAMTS2*) were designed based on available sequences using the Primer-BLAST suite (details in Supplementary Table S4). Whenever possible, primers were located in different exons or at an exon–exon junctions to minimize inaccuracies due to genomic DNA contamination. For each primer pair, a preliminary qRT-PCR assay, on a bulk of samples, was performed to check for amplification of non-specific products or primer-dimer artifacts and efficiency (E) were assessed. The qRT-PCR reaction was carried out aliquoting 5 *μ*l of a five-fold diluted cDNA and SsoFast™ EvaGreen® Supermix (BioRad). The amplification was performed in a CFX96 Touch instrument (BioRad, Hercules, CA) with the following conditions: 98 °C for 3 min, then 50 cycles of 98 °C for 10 s and 60 °C for 15″. Fluorescence data were collected at the end of the second step and, following cycling, the melting curve was determined in the range of 58–95 °C with an increment of 0.01 °C/sec. Each reaction was run in triplicate with appropriate negative controls.

### Data analyses

Data analysis was carried out with Bio-Rad CFX Manager software (ver. 3.2.2) and GenEx (ver.6). To analyze gene expression stability of HKGs, geNorm algorithm, included on CFX Manager software (ver. 3.2.2), was used^35^. The expression ratio of the genes of interest was normalized relative to the abundance of the two reference genes (*HBS2*, *GUS*β) using the ΔΔCq method. Samples were divided in two groups, melanomas (MM) and melanocytomas (MCT) and were tested for parametric data distribution using the Kolmogorov-Smirnov (KS) Test. Changes in the relative gene expression between groups were calculated using Mann-Whitney Test for unpaired data, because the data were not-normally distributed. All expression values are presented as means in a base 2 logarithmic scale with a confidence interval of 95%.

### Immunohistochemical examination

For immunohistochemical analysis, paraffin blocks were cut into sections of 5 µm thickness, mounted on positively charged slides, dewaxed and rehydrated. Immunohistochemistry was performed using rabbit polyclonal antibodies against Collagen 1 (COL1, Biorbyt, San Francisco, CA) and Thrombospondin 2 (THBS2, Biorbyt, San Francisco, CA). Antigen retrieval was performed in a microwave oven with citrate buffer (pH 6.0). Blocking of endogenous peroxidase with 3% H_2_O_2_ solution for 10 minutes at room temperature was performed. Primary antibodies were incubated overnight at 4 °C (dilution 1:100 for COL1; 1:200 for THBS2). Then, slides were treated with a ready-to-use kit (Mouse and Rabbit Specific HRP; Abcam, Cambridge, UK) following the manufacturer’s instructions. Positive reactions were revealed with 3-amino-9-ethilcarbazole (AEC Substrate; Abcam, Cambridge, UK). Positive cells were blindly evaluated by two operators (CB, IP) on the total tumoral section and assigned a percentage value (0–100% representing the percentage of positive neoplastic cells on the total number of neoplastic cells), an intensity (1 = mild, 2 = moderate, 3 = marked), a pattern (peripheral, diffuse, random) and distribution (focal, multifocal, diffuse). Differences between groups were evaluated with a two tailed Student t-test for non paired samples. All statistical calculations were performed using R base statistical package. A p value lower than 0.05 was set as threshold for statistical significance. Other proteins corresponding to the target genes of the qRT-PCR could not be analysed, due to the unavailability of specific anti-canine antibodies for formalin-fixed, paraffin-embedded samples.

## Electronic supplementary material

Supplementary Table 1, 2 and 4

Supplementary Table 3
