# Supplementary material for: Transcriptome Analysis of Canine Cutaneous Melanoma and Melanocytoma Reveals a Modulation of Genes Regulating Extracellular Matrix Metabolism and Cell Cycle
Source: Sci Rep. 2017 Jul 25;7:6386. doi: 10.1038/s41598-017-06281-1 (PMC5526991; doi:10.1038/s41598-017-06281-1)
Supplement: Supplementary file 1 — Supplementary Table 1, 2 and 4 [file 41598_2017_6281_MOESM1_ESM.pdf]

TRANSCRIPTOME ANALYSIS OF CANINE CUTANEOUS MELANOMA AND MELANOCYTOMA REVEALS A MODULATION OF GENES REGULATING EXTRACELLULAR MATRIX METABOLISM AND CELL CYCLE

Chiara Brachelente, Katia Cappelli, Stefano Capomaccio, Ilaria Porcellato, Serenella Silvestri, Laura Bongiovanni, Raffaella De Maria, Andrea Verini Supplizi, Luca Mechelli & Monica Sforna

Supplementary Table 1. Summary statistics on the sequenced samples in read pairs of samples submitted to RNA-seq.

Case No.	Raw Reads	QC Passed	Discarded Seqs (%)	Uniquely Mapped	Uniquely Mapped %
**1**	33.934.336	33.701.111	0,69	27.971.790	83,00%
**4**	39.249.148	38.781.813	1,19	6.515.145	16,80%
**5**	70.144.356	69.180.820	1,37	10.829.792	15,65%
**6**	31.183.354	30.938.657	0,78	17.684.984	57,16%
**13**	23.794.350	23.611.093	0,77	17.364.063	73,54%
**16**	27.417.386	27.308.707	0,40	25.489.959	93,34%
**17**	33.658.370	33.528.972	0,38	29.011.664	86,53%
**18**	28.632.604	28.544.089	0,31	26.615.786	93,24%

Supplementary Table 2. Differentially expressed genes ranked by fold change. Annotation was added with Biomart.

Ensembl Gene ID	logFC	PValue	FDR (qValue)	Gene Name	Description
**ENSCAFG00000029553**	4.852	1.60E-06	1.27E-03		
**ENSCAFG00000019985**	4.580	2.15E-05	7.90E-03	***COL11A1***	collagen, type XI, alpha 1
**ENSCAFG00000000903**	4.365	1.23E-05	5.56E-03	***COL11A2***	collagen, type XI, alpha 2
**ENSCAFG00000010530**	4.306	2.14E-10	1.85E-06	***CCDC80***	coiled-coil domain containing 80
**ENSCAFG00000005603**	3.990	8.44E-09	1.99E-05	***ISM1***	isthmin 1 homolog
**ENSCAFG00000017618**	3.907	3.33E-07	3.27E-04	***DRP2***	dystrophin related protein 2
**ENSCAFG00000007036**	3.790	2.34E-05	8.36E-03	***CD22***	CD22 molecule
**ENSCAFG00000031675**	3.687	2.13E-05	7.90E-03	***COL8A1***	collagen, type VIII, alpha 1
**ENSCAFG00000017018**	3.621	7.80E-10	3.06E-06	***COL1A1***	collagen, type I, alpha 1
**ENSCAFG00000018642**	3.442	5.03E-005	0.014784381	***NOS2***	nitric oxide synthase
**ENSCAFG00000000874**	3.230	3.20E-08	5.38E-05	***THBS2***	thrombospondin-2
**ENSCAFG00000023774**	3.197	9.67E-005	0.023694412	***RAMP1***	receptor (G protein-coupled) activity modifying protein 1
**ENSCAFG00000012718**	3.179	2.49E-07	2.70E-04	***CPXM2***	carboxypeptidase X, member 2
**ENSCAFG00000018761**	3.178	9.69E-07	8.77E-04	***PIEZO2***	piezo-type mechanosensitive ion channel component 2
**ENSCAFG00000005527**	3.106	5.74E-06	3.24E-03	***P4HA3***	prolyl 4-hydroxylase, alpha polypeptide III
**ENSCAFG00000002069**	3.093	3.14E-10	1.85E-06	***COL1A2***	collagen, type I, alpha 2
**ENSCAFG00000000868**	3.075	2.52E-07	2.70E-04	***SMOC2***	SPARC related modular calcium binding 2
**ENSCAFG00000006865**	2.943	3.62E-05	1.15E-02	***PAMR1***	peptidase domain containing associated with muscle regeneration 1
**ENSCAFG00000002307**	2.933	2.15E-06	1.49E-03	***ASPN***	asporin
**ENSCAFG00000014812**	2.898	4.20E-08	6.18E-05	***COL3A1***	collagen, type III, alpha 1
**ENSCAFG00000002947**	2.896	0.000230822	0.046828998	***PRSS35***	protease, serine, 35

**ENSCAFG00000005843**	2.892	0.000210632	0.043482483	***LTBP1***	latent transforming growth factor binding protein 1
**ENSCAFG00000000667**	2.890	4.17E-06	2.58E-03	***FNDC1***	fibronectin type III domain containing 1
**ENSCAFG00000010930**	2.851	1.97E-09	5.78E-06	***COL16A1***	collagen, type XVI, alpha 1
**ENSCAFG00000009282**	2.751	8.78E-06	4.49E-03	***CREB3L1***	cAMP responsive element binding protein 3-like 1
**ENSCAFG00000005880**	2.742	7.33E-005	0.019612931		
**ENSCAFG00000006608**	2.699	1.94E-06	1.42E-03	***CPXM1***	carboxypeptidase X, member 1
**ENSCAFG00000014837**	2.635	7.01E-08	9.16E-05	***COL5A2***	collagen, type V, alpha 2
**ENSCAFG00000019238**	2.605	5.79E-06	3.24E-03	***MMP23B***	matrix metallopeptidase 23B
**ENSCAFG00000020166**	2.517	2.67E-08	5.23E-05	***ANGPTL2***	angiopoietin-like 2
**ENSCAFG00000032273**	2.498	0.000182255	0.04046415		
**ENSCAFG00000012762**	2.482	0.00012663	0.029521978	***COCH***	coagulation factor C homolog, cochlin
**ENSCAFG00000011908**	2.462	7.06E-06	3.77E-03	***SHTN1***	shootin 1
**ENSCAFG00000013079**	2.445	4.06E-06	2.58E-03	***OLFML2B***	olfactomedin-like 2B
**ENSCAFG00000019848**	2.443	1.09E-05	5.14E-03	***COL5A1***	collagen, type V, alpha 1
**ENSCAFG00000020367**	2.428	0.00019811	0.042384719	***PLEKHG4***	pleckstrin homology domain containing, family G member 4
**ENSCAFG00000028461**	2.397	9.82E-06	4.82E-03	***LOX***	lysyl oxidase
**ENSCAFG00000006424**	2.389	4.27E-05	1.32E-02	***GXYLT2***	glucoside xylosyltransferase 2
**ENSCAFG00000008056**	2.370	6.47E-005	0.018125915	***RYR3***	ryanodine receptor 3
**ENSCAFG00000010061**	2.344	7.70E-005	0.020142478	***ABCC11***	ATP-binding cassette, sub-family C, member 11
**ENSCAFG00000000686**	2.315	0.000127953	0.029521978		
**ENSCAFG00000014670**	2.247	4.61E-05	1.39E-02	***NID2***	nidogen 2 (osteonidogen)
**ENSCAFG00000016828**	2.142	2.84E-05	9.43E-03	***COPZ2***	coatomer protein complex, subunit zeta 2
**ENSCAFG00000017262**	2.136	2.88E-05	9.43E-03	***MYH10***	myosin, heavy chain 10

**ENSCAFG00000006234**	2.081	6.81E-005	0.018646387	***ADAM33***	ADAM metallopeptidase domain 33
**ENSCAFG00000010387**	2.055	1.62E-06	1.27E-03	***FAP***	fibroblast activation protein, alpha
**ENSCAFG00000019943**	2.051	2.59E-05	8.95E-03	***CRISPLD2***	cysteine-rich secretory protein LCCL domain containing 2
**ENSCAFG00000001792**	2.033	0.000100326	0.024092502	***LIMCH1***	LIM and calponin homology domains 1
**ENSCAFG00000003044**	2.013	0.000208688	0.043482483	***FKBP14***	FK506 binding protein 14
**ENSCAFG00000008341**	2.001	0.000243386	0.048256918	***SPON1***	Uncharacterized protein
**ENSCAFG00000006073**	1.971	0.000246062	0.048256918	***POSTN***	periostin
**ENSCAFG00000013256**	1.957	1.46E-05	5.94E-03	***MRC2***	mannose receptor, C type 2
**ENSCAFG00000012226**	1.931	9.66E-005	0.023694412	***COL6A3***	collagen, type VI, alpha 3
**ENSCAFG00000001875**	1.876	8.97E-005	0.022950061	***CKAP4***	cytoskeleton-associated protein 4
**ENSCAFG00000000334**	1.849	1.84E-05	7.22E-03	***ADAMTS2***	ADAM metallopeptidase with thrombospondin type 1 motif, 2
**ENSCAFG00000014322**	1.661	6.44E-005	0.018125915	***PCOLCE***	procollagen C-endopeptidase enhancer
**ENSCAFG00000018570**	-2.306	0.00018037	0.04046415	***SGIP1***	SH3-domain GRB2-like (endophilin) interacting protein 1
**ENSCAFG00000023349**	-4.142	1.44E-05	5.94E-03		
**ENSCAFG00000011838**	-5.263	1.40E-05	5.94E-03	***ST8SIA1***	ST8 alpha-N-acetyl-neuraminide alpha-2,8-sialyltransferase 1
**ENSCAFG00000012848**	-6.947	0.000185903	0.040509549	***COL20A1***	collagen, type XX, alpha 1

Supplementary Table 4. Primer were designed based on available sequences using the Primer-BLAST software.

Gene	Accession	Forward	Reverse	Efficiency	Amp. Size
GUSβ	NM_001003191	GTGCTGGATCAGAAACGCAA	CTTTGGGTTGTCTCTGGCGA	96%	136
HMBS	XM_546491.5	GGTGGGCGTGTTGCATGA	AGGTACAGTTGCCCATCCTTCATAG	105%	132
THBS2	NM_001197156	ATTCGCCATCAAGGCAAGGA	CCACGGACCCAAACTCATCA	110%	85
COL1A1	NM_001003090	AGAGCATGACCGACGGATTC	ACGCTGTTCTTGCAGTGGTA	90	139
COL11A1	XM_005621852	AAGCTCAAGCTCAGGCGATT	TCACCACTCTCACCTTTGGC	87%	133
NOS2	NM_001313848	CCTACCAGTACGACTTGACCG	TGGGGTCATCCTGTGTCACT	99%	96
ADAMTS2	XM_843844	GTGGAGTGGGAGTACAGGGA	GTATGTCAGCGAGATCCGGG	113%	120
